# MiR-578 and miR-573 as potential players in BRCA-related breast cancer angiogenesis

**DOI:** 10.18632/oncotarget.2509

**Published:** 2014-09-25

**Authors:** Katia Danza, Simona De Summa, Rosamaria Pinto, Brunella Pilato, Orazio Palumbo, Giuseppe Merla, Gianni Simone, Stefania Tommasi

**Affiliations:** ^1^ IRCCS “Giovanni Paolo II”, Molecular Genetics Laboratory – Bari, Italy; ^2^ IRCCS Casa Sollievo della Sofferenza, Medical Genetics Unit – San Giovanni Rotondo (FG), Italy; ^3^ IRCCS “Giovanni Paolo II”, Anatomopathology Unit – Bari, Italy

**Keywords:** miR-573, miR-578, BRCA, familial breast cancer, angiogenesis

## Abstract

The involvement of microRNA (miRNAs), a new class of small RNA molecules, in governing angiogenesis has been well described. Our aim was to investigate miRNA-mediated regulation of angiogenesis in a series of familial breast cancers stratified by BRCA1/2 mutational status in BRCA carriers and BRCA non-carriers (BRCAX). Affymetrix GeneChip miRNA Arrays were used to perform miRNA expression analysis on 43 formalin-fixed paraffin-embedded (FFPE) tumour tissue familial breast cancers (22 BRCA 1/2-related and 21 BRCAX). Pathway enrichment analysis was carried out with the DIANA miRPath v2.0 web-based computational tool, and the miRWalk database was used to identify target genes of deregulated miRNAs. An independent set of 8 BRCA 1/2-related and 11 BRCAX breast tumors was used for validation by Real-Time PCR. *In vitro* analysis on HEK293, MCF-7 and SUM149PT cells were performed to best-clarify miR-573 and miR-578 role. A set of 16 miRNAs differentially expressed between BRCA 1/2-related and BRCAX breast tumors emerged from the profile analysis. Among these, miR-578 and miR-573 were found to be down-regulated in BRCA 1/2-related breast cancer and associated to the Focal adhesion, Vascular Endothelial Growth Factor (VEGF) and Hypoxia Inducible Factor-1 (HIF-1) signaling pathways. Our data highlight the role of miR-578 and miR-573 in controlling BRCA 1/2-related angiogenesis by targeting key regulators of Focal adhesion, VEGF and HIF-1 signaling pathways.

## INTRODUCTION

Angiogenesis is the formation of new vessels from pre-existing ones and is a necessary step for cancer growth and progression. As result of an imbalance between pro- and antiangiogenic signals, the primitive vasculature expands into a new complex network [1]. The involvement of epigenetic mechanisms in the regulation of angiogenesis has been previously described [2]. MicroRNAs are small, non-coding RNAs that control numerous cellular pathways through the regulation of gene expression at the post-transcriptional level [3]. Due to their oncogenic and oncosuppressor properties, miRNAs can act as both pro-angiogenic or anti-angiogenic elements during the angiogenic switch [2]. A pivotal role for miRNAs has been well-described in breast tumors as regulators of angiogenesis [4] but, to our knowledge, no data are available when the focus is restricted to familial breast tumor subgroup. BRCA1 and BRCA2 are the best-known cancer susceptibility genes associated with the hereditary breast tumors. Inheritance of BRCA genes mutations increases the lifetime risk of breast and ovarian cancer development [5]. A functional link between BRCA1 and miRNAs has been recently observed [6] but very little is known about miRNAs involvement in familial breast cancer with and without BRCA mutations [7-11]. The ability of BRCA1 to regulate miRNA expression and in turn, the capacity of several miRNAs to down-regulate BRCA1 [12] suggests a novel way through which BRCA1 can play a pivotal role in several processes, including vascular remodeling. The involvement of BRCA1 in neovascularization has been well-demonstrated [13-16]. Our previous study reported increased levels of both angiopoietin-1/-2 and VEGF in BRCA-related breast tumors, highlighting their contribution to vascular remodeling in patients harboring BRCA1/2 mutations [17]. Moreover, a possible deregulation in BRCA-related angiogenesis and hypoxia signaling pathways was suggested by changes in VEGF [18] and in hypoxia-related HIF-1 alpha [18-21] protein levels with respect to BRCA status. Given BRCA1 and miRNAs crosstalk [6,12], we supposed that BRCA1 contribution to neovascularization and hypoxia response could also involve many epigenetic mechanisms. As miRNAs have become a key concept in tumor vascular regulation, our aim was to explore their impact on angiogenic and hypoxic signaling pathways in familial breast cancer with respect to BRCA mutational status. Our data highlighted the involvement of miR-573 and miR-578 in the VEGF, Focal Adhesion Kinase (FAK) and HIF-1 signaling pathways, highlighting their role in BRCA1/2-related breast tumor angiogenesis.

## RESULTS

### MiRNA expression analysis in familial breast cancer

**Figure 1:**
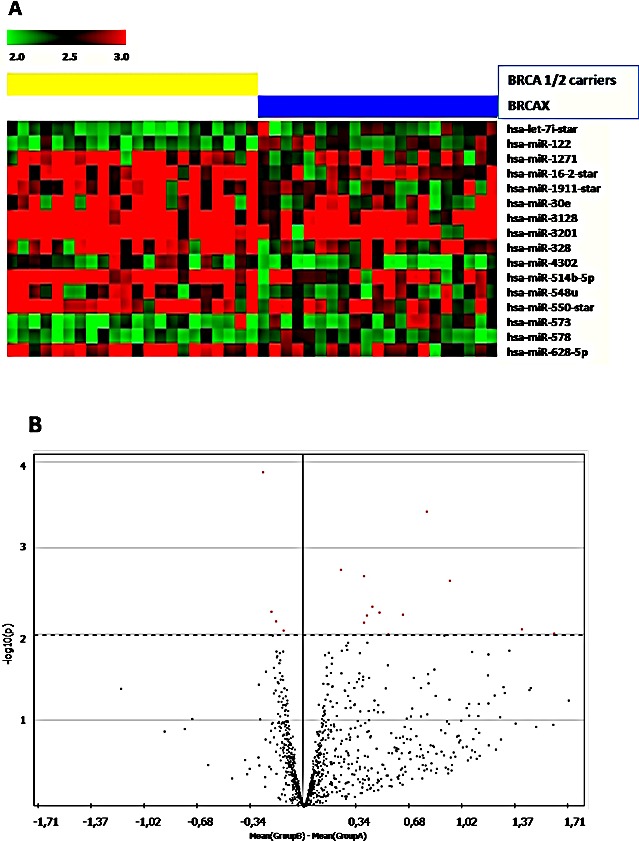
(A) Heatmap showing miRNAs significantly (p<0.001) deregulated in BRCA1/2-related breast (left) tumors and BRCAX (right). Red dots represent up-regulated and green down-regulated miRNAs. (B) Volcano plot, an intuitive way to visualize differences in mean fluorescence in relation to p-value. Significant miRNAs are located above the dotted line, which indicate −log_10_[cut-off p-value < 0.01 considered in the statistical analysis. Left side: miRNAs which are down-regulated in BRCA1/2 mutation carriers; right side: up-regulated miRNAs in BRCA1/2 mutation carriers.

MiRNA expression profiling was performed on a training set of 43 FFPE familial breast cancer cases divided into 22 BRCA1/2-related and 21 BRCAX. We selected miRNAs annotated as “hsa” in order to exclusively analyze the differential expression of human genes. The selected hsa-miRNAs (n=1100) underwent statistical analysis through the t-test. The microarray analysis revealed 16 miRNAs significantly differentially modulated (p<0.01) between BRCA1/2 carriers and breast tumors without BRCA1/2 mutations, as reported in the heatmap (Figure 1A) and in the volcano plot (Figure 1B). It could be preliminary observed that only 4 (miR-let7i_star, miR-122, miR-573 and miR-578) out of 16 deregulated miRNAs were decreased in BRCA1/2-related breast tumors, whereas all others were found to be up-regulated, as confirmed by the mean fluorescence values reported in Table 1.

**Table 1:** Mean intensity level of fluorescence of miRNAs differentially expressed between BRCAX and BRCA1/2-related breast tumors with a statistical significance p<0.01

miRNA	Mean BRCAX breast cancer	Mean BRCA1/2- related breast cancer	p-value
**hsa-let-7i-star**	2.427	2.247	0.006911965
**hsa-miR-122**	2.518	2.253	0.000127841
**hsa-miR-1271**	2.629	3.017	0.002058682
**hsa-miR-16-2-star**	2.741	3.681	0.002333036
**hsa-miR-1911-star**	2.548	3.185	0.00581033
**hsa-miR-30e**	2.687	3.231	0.009791313
**hsa-miR-3128**	3.656	5.061	0.008587457
**hsa-miR-3201**	4.341	5.954	0.009598941
**hsa-miR-328**	2.652	3.057	0.005943566
**hsa-miR-4302**	2.228	2.467	0.00174379
**hsa-miR-514b-5p**	2.758	3.549	0.000368078
**hsa-miR-548u**	2.455	2.840	0.007214292
**hsa-miR-550-star**	2.772	3.259	0.005490081
**hsa-miR-573**	2.428	2.217	0.005391205
**hsa-miR-578**	2.357	2.224	0.008889099
**hsa-miR-628-5p**	2.678	3.119	0.004701929

### Pathway Enrichment analysis

**Figure 2:**
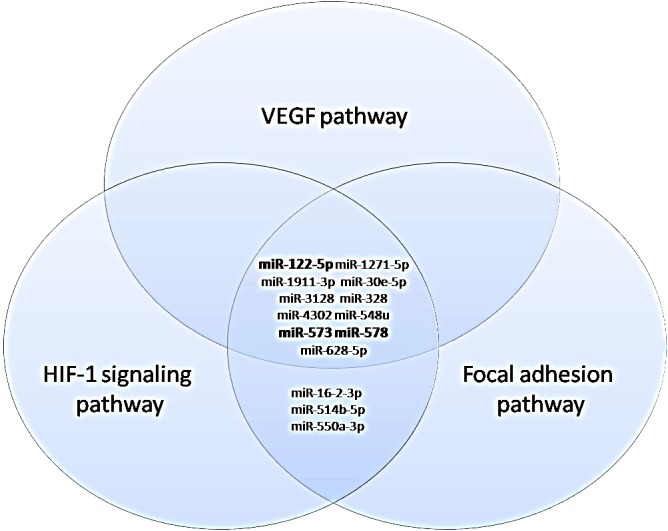
Venn diagrams representing commonly deregulated miRNAs in the VEGF, Focal adhesion and HIF-1 signaling pathways. In bold are shown down-regulated miRNAs common to the three angiogenic signaling pathways.

To investigate whether the co-expression of the 16 deregulated miRNAs could affect angiogenic signaling in BRCA1/2-related tumors by targeting angiogenic factors, the Diana mir-path web-based computational tool was used. Table 2 shows the results from the KEGG pathway enrichment analysis (p<0.05). The set of deregulated miRNAs seemed to be associated with 80 diverse signaling cascades. Among these, we focused on the VEGF, Focal Adhesion and HIF-1 pathways as they are all involved in angiogenesis process. Interestingly, 11 out of 16 deregulated miRNAs were linked to the VEGF signaling cascade by targeting 19 diverse genes, whereas 14 out of 16 deregulated miRNAs were associated to focal adhesion and hypoxia pathways by targeting 60 and 32 diverse genes, respectively (Table 3). Since our previous study [17] reported higher levels of key angiogenic markers in BRCA1/2 carriers, we primarily focused on miRNAs associated to angiogenic signaling pathways and down-regulated in BRCA-related tumors. It was interesting to note that among these, miR-122, miR-578 and miR-573 were commonly associated to the VEGF, Focal Adhesion and HIF-1 signaling pathways and thus used for further analysis (Figure 2).

**Table 2:** Pathways linked to the 16 significantly deregulated miRNAs

KEGG pathway	N° of miRNA	p-value	N° of target genes
Long-term potentiation	13	6.84E-22	33
ErbB signaling pathway	12	1.16E-15	35
Dilated cardiomyopathy	13	5.34E-14	36
ABC transporters	8	5.27E-12	20
Prostate cancer	13	2.15E-11	33
Glutamatergic synapse	13	1.71E-10	40
Neurotrophin signaling pathway	13	3.43E-10	42
Regulation of actin cytoskeleton	14	3.90E-10	65
Arrhythmogenic right ventricular cardiomyopathy [ARVC]	13	5.59E-10	31
Focal adhesion	14	3.69E-09	60
Hypertrophic cardiomyopathy [HCM]	12	5.80E-09	30
Axon guidance	13	9.22E-09	46
Ubiquitin mediated proteolysis	12	1.07E-08	45
Glioma	13	1.69E-08	27
Insulin signaling pathway	14	1.69E-08	43
Endometrial cancer	12	4.55E-07	20
B cell receptor signaling pathway	11	5.96E-07	26
Non-small cell lung cancer	12	7.85E-07	20
PI3K-Akt signaling pathway	14	1.36E-06	86
GnRH signaling pathway	12	1.80E-06	29
Progesterone-mediated oocyte maturation	12	1.80E-06	28
mTOR signaling pathway	12	2.29E-06	22
Endocytosis	12	2.84E-06	56
Acute myeloid leukemia	10	3.72E-06	20
Pancreatic cancer	13	4.62E-06	24
Amyotrophic lateral sclerosis	8	5.70E-06	18
Phosphatidylinositol signaling system	12	9.39E-06	29
Fc gamma R-mediated phagocytosis	12	1.80E-05	29
Calcium signaling pathway	12	1.87E-05	50
Bacterial invasion of epithelial cells	11	1.89E-05	24
Endocrine and other factor-regulated calcium reabsorption	7	1.97E-05	18
Dopaminergic synapse	14	1.97E-05	39
Small cell lung cancer	14	4.85E-05	26
TGF-beta signaling pathway	13	5.27E-05	25
Shigellosis	11	5.54E-05	21
Cholinergic synapse	14	6.70E-05	36
Gap junction	10	7.56E-05	26
Chronic myeloid leukemia	12	7.56E-05	24
Melanoma	11	7.66E-05	23
HIF-1 signaling pathway	14	7.66E-05	32
Colorectal cancer	11	7.84E-05	21
Long-term depression	11	0.00010297	22
Type II diabetes mellitus	8	0.000254913	16
MAPK signaling pathway	13	0.000254913	67
Oocyte meiosis	12	0.00028102	36
Gastric acid secretion	12	0.00028102	23
Inositol phosphate metabolism	10	0.000438238	20
Natural killer cell mediated cytotoxicity	12	0.000438238	39
Vascular smooth muscle contraction	13	0.00054281	34
Aldosterone-regulated sodium reabsorption	10	0.000675186	13
Pathways in cancer	15	0.000805191	88
Melanogenesis	12	0.001443074	27
RNA degradation	10	0.001527123	21
Taurine and hypotaurine metabolism	4	0.002484891	4
Renal cell carcinoma	11	0.002484891	22
Retrograde endocannabinoid signaling	13	0.002555835	32
T cell receptor signaling pathway	11	0.002611728	29
VEGF signaling pathway	11	0.002830415	19
Glycosylphosphatidylinositol[GPI]-anchor biosynthesis	6	0.003458651	9
Serotonergic synapse	11	0.004099713	30
Amphetamine addiction	11	0.004449922	23
Propanoate metabolism	7	0.005323316	10
GABAergic synapse	12	0.005836946	28
Protein processing in endoplasmic reticulum	14	0.00600624	43
Wnt signaling pathway	14	0.006229163	42
Cell cycle	12	0.006354843	33
Chemokine signaling pathway	14	0.006702432	45
Glycerophospholipid metabolism	12	0.008604037	26
Biotin metabolism	1	0.01053896	1
Carbohydrate digestion and absorption	9	0.01053896	12
Hepatitis B	14	0.01246892	40
Circadian rhythm	9	0.01363142	10
Osteoclast differentiation	13	0.01363142	33
Tight junction	13	0.01376918	35
Transcriptional misregulation in cancer	14	0.0144959	46
mRNA surveillance pathway	12	0.0145927	23
Adherens junction	12	0.03073926	23
Viral myocarditis	12	0.03463523	18
N-Glycan biosynthesis	10	0.03514185	15
Salmonella infection	12	0.04015256	21

**Table 3:** Focal Adhesion, HIF-1 and VEGF signaling pathways linked to the deregulated miRNAs

KEGG pathway	N° of miRNA	miRNA	N° of target genes	target genes
**Focal adhesion pathway**	14	hsa-miR-122-5p hsa-miR-1271-5p hsa-miR-16-2-3p hsa-miR-1911-3p hsa-miR-30e-5p hsa-miR-3128 hsa-miR-328 hsa-miR-4302hsa-miR-514b-5p hsa-miR-548u hsa-miR-550a-3phsa-miR-628-5p hsa-miR-578 hsa-miR-573	60	ACTB TLN2 GSK3B PDGFRA ACTN2 ERBB2 ROCK1 ITGA9 ITGA8 PIK3CB CAV1 PIK3R2 RAP1A ROCK2 ITGA5 ITGA3 IGF1R EGFR CAV2 ITGA1 FYN SHC3 PTK2 PPP1R12A PIK3CD PIK3R3 DOCK1 MAPK8 ITGB1 PARVA RELN FLNB ACTN1 FLT1 PDK1 FLNA ITGA2 COL4A4 SOS1 ITGA10 RAC1 PRKCB LAMC1 SHC4 AKT3 PDGFC MYLK3 COL11A1 PIK3CA ITGA4 ITGA6 VAV3 PTEN MAPK1 RAP1B MYLK ILK COL4A1 ITGB3 PDGFA
**HIF-1 signaling pathway**	14	hsa-miR-122-5p hsa-miR-1271-5p hsa-miR-16-2-3p hsa-miR-1911-3p hsa-miR-30e-5p hsa-miR-3128 hsa-miR-328 hsa-miR-548u hsa-miR-4302 hsa-miR-514b-5p hsa-miR-550a-3p hsa-miR-573 hsa-miR-578 hsa-miR-628-5p	32	CAMK2D ERBB2 CAMK2G PIK3CB CUL2 IFNGR2 PIK3R2 ANGPT2 IGF1R EGFR RPS6KB2 CAMK2A PIK3CD PIK3R3 EIF4E FLT1 CYBB PRKCB AKT3 PIK3CA IFNG SLC2A1 MKNK1 PFKFB2 EDN1 MTOR ALDOA MAPK1 CREBBP RPS6KB1 EGLN1 PFKFB3
**VEGF signaling pathway**	11	hsa-miR-122-5p hsa-miR-1271-5p hsa-miR-1911-3p hsa-miR-30e-5p hsa-miR-3128 hsa-miR-328 hsa-miR-4302 hsa-miR-548u hsa-miR-573 hsa-miR-578 hsa-miR-628-5p	19	NRAS PIK3CB PIK3R2 KRAS PPP3CA PTK2 NFAT5 PIK3CD PIK3R3 PPP3CB NFATC2 RAC1 PPP3R1 PRKCB AKT3 PIK3CA NFATC3 MAPK1 PPP3R2

Computational analysis emphasized VEGFA, HIF1A (DIANAmt, miRanda, miRDB, miRWALK, PICTAR5, TargetScan], ANGPT2 [miRanda, PICTAR5, TargetScan) and FAK (miRanda, TargetScan, PICTAR5) as predictive targets of miR-578. Furthermore, FAK (miRanda, TargetScan, PICTAR5), VEGFA (RNA22) and ANGPT2 (miRanda, miRDB, miRWALK, TargetScan) also resulted as potential targets of miR-573, whereas VEGFA as a validated target of miR-122.

### Microarray analysis validation by quantitative real time PCR

**Figure 3:**
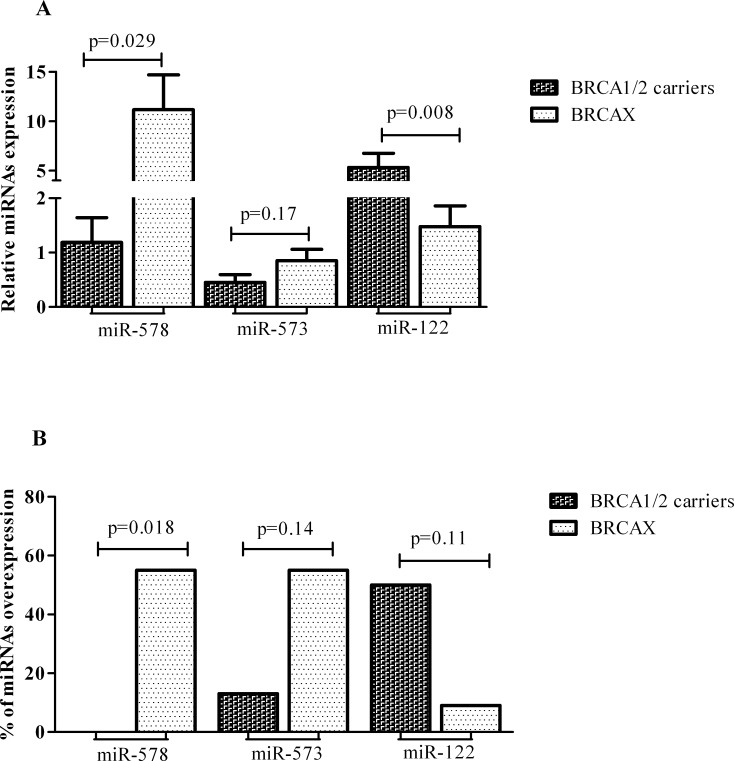
MiR-578, miR-573 and miR-122 in BRCA1/2 mutation carriers and BRCAX associated tumors: (A) Mean levels with S.E.M. (B) Percentage of the overexpression.

Mir-578, miR-573 and miR-122 were selected for validation on an independent set of 19 FFPE familial breast tumors subdivided into 8 BRCA1/2 related breast cancer cases and 11 BRCAX associated breast tumors. Quantitative real time PCR confirmed the down-expression of miR-578 and miR-573 in BRCA1/2 carriers. On the contrary, a reverse trend compared to microarray analysis was reported for miR-122 expression in familial breast tumors with and without BRCA1/2 mutations (Figure 3A). The mean expression level of miR-578 was significantly lower in patients harboring BRCA1/2 germline mutations compared to the BRCAX breast tumor group (1.18 vs 11.16, p=0.029), as was the mean level of miR-573, although this did not reach the statistical significance (0.45 vs 0.84 p=0.17). On the contrary, higher mean levels of miR-122 were reported in BRCA1/2 carriers compared to BRCAX (5.316 vs 1.477, p=0.008). In addition, the percentage of miR-578, miR-573 and miR-122 overexpression in BRCA1/2 carriers and BRCAX associated tumors was also explored using the tumor mean level as cut-off (Figure 3B). A lower frequency of miR-578 and miR-573 overexpression was observed in BRCA1/2-related breast tumors compared to BRCAX breast cancers (0% vs 55%, p=0.018 and 13% vs 55%, p=0.14), respectively. On the contrary, miR-122 was more frequently overexpressed in BRCA1/2 carriers compared to BRCAX (50% vs 9% p=0.11).

### Expression analysis of VEGFA, FAK, HIF1A and ANGPT2 genes in the validation set

**Figure 4:**
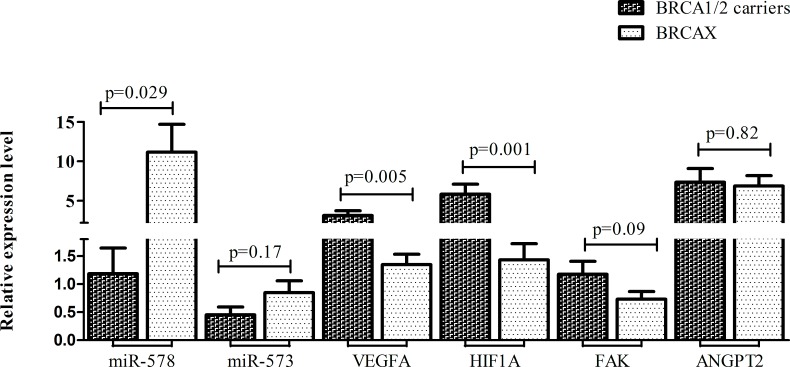
Mean expression levels with S.E.M. of both miR-578, miR-573 and their target genes VEGFA, HIF1A, FAK and ANGPT2 in BRCA1/2 carriers and BRCAX associated tumors.

VEGFA gene expression, a putative target of miR-578 and miR-573, was explored. The mean expression level of this gene was higher in BRCA1/2 carriers (n=8) compared to BRCAX (n=11) tumors (VEGFA: 3.13 vs 1.34, p=0.005). Moreover, the mRNA level of FAK, ANGPT2 and HIF1A has been investigated. All three genes presented higher mean expression levels in BRCA1/2 carriers compared to BRCAX tumors (HIF1A: 5.82 vs 1.43, p=0.001; FAK: 1.17 vs 0.73, p=0.099; ANGPT2: 7.35 vs 6.86, p= 0.82) (Figure 4). Furthermore, BRCA1/2-related breast cancers also presented a higher percentage of VEGFA, HIF1A, FAK and ANGPT2 gene overexpression compared to the BRCAX breast tumour group (VEGFA: 62% vs 9%, p=0.04; HIF1A: 62% vs 0%, p=0.004; FAK: 62% vs 18%, p=0.073; ANGPT2: 50% vs 45%, p= 1.00).

In order to verify the putative association between angiogenic markers (VEGFA, HIF1, FAK and ANGPT2) and miR-578 or miR-573, a correlation analysis was performed on the entire cohort. MiR-578 negatively correlated with each angiogenic factor (VEGFA: r=-0.3, p=0.19; HIF1A: r=-0.45, p=0.05; FAK: r=-0.26, p=0.27; ANGPT2: r=-0.33, p=0.16), although a statistical significance was reached only for HIF1A. On the contrary, miR-573 negatively correlated only with VEGFA (r=-0.16, p=0.5) and ANGPT2 (r=-0.27, p=0.25).

### MiR573 and miR578 regulation of VEGFA, FAK, HIF1A and ANGPT2 in *in vitro* model

**Figure 5:**
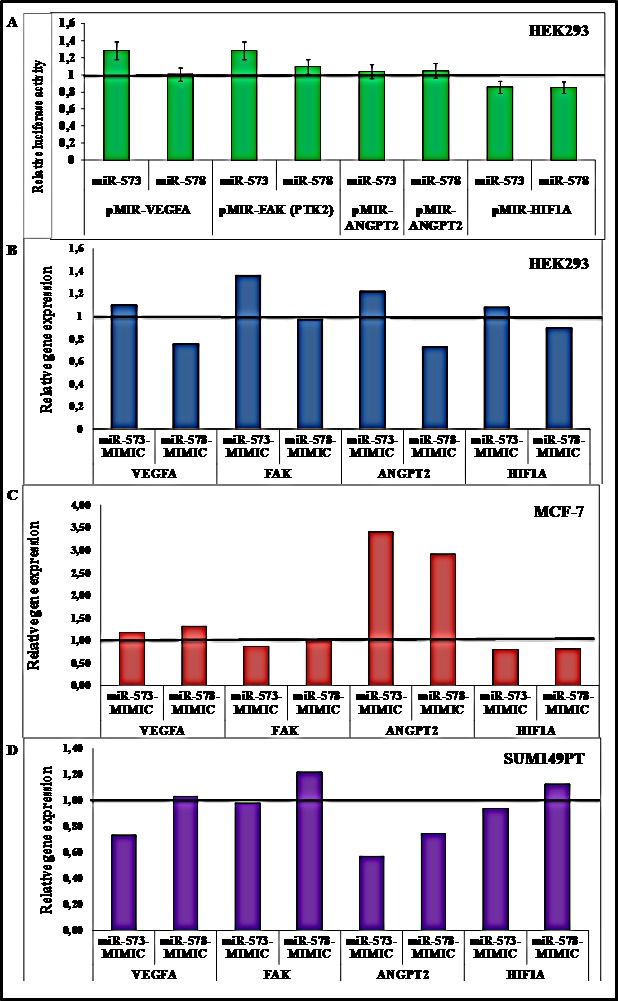
(A) HEK293 cells were co-transfected with the indicated reporter constructs. The Luciferase Activity was normalized to the level of Renilla luciferase (pRNL) and the Relative Luciferase Activity (RLA) was calibrated to 1 that refers the RLA of cells transfected with the hsa-miR-negative control as reported in Materials and Methods; cells were transfected with hsa-miR-578, hsa-miR-573 and negative control miRNA mimic (B) HEK293 (C) MCF-7 and (D) SUM149PT. Cells transfected with the negative control were calibrated to 1 and relative expression of VEGFA, FAK, ANGPT2 and HIF1A genes was explored.

To experimentally test whether miR-573 and miR-578 directly targets the 3′UTR of VEGFA, FAK, ANGPT2 and HIF1A *in vitro*, HEK293 cells were co-transfected with reporter constructs along with a synthetic mimic of miR-573 or miR-578. As reported in Figure 5A, the overexpression of the indicated miRNAs did not affect the luciferase activity of the reporter construct when compared to the control, indicating that miR-573 and miR-578 do not regulate VEGFA, FAK, ANGPT2 and HIF1A expression directly binding to the 3′UTR region of the genes.

Subsequently, to verify the hypothesis of an indirect epigenetic regulation, mRNA levels of VEGFA, FAK, ANGPT2 and HIF1A were explored in HEK293, MCF-7 and SUM149PT cells transfected with hsa-miR-578, hsa-miR-573 and negative control miRNA mimic.

Using HEK293 as an *in vitro* model, hsa-miR-578 mimic transfected cells showed a lower expression of HIF1A, VEGFA and ANGPT2 genes when compared to the control, whereas no reduction in the three mRNA levels was observed for hsa-miR-573 mimic transfected cells (Figure 5B). As reported in Figure 5C, both hsa-miR-578 and hsa-miR-573 mimic MCF-7 transfected cells showed lower HIF1A levels whereas hsa-miR-573 mimic transfected cells also showed FAK reduced expression. In BRCA1 mutated cell line SUM149PT, hsa-miR-573 mimic transfection lead to a lower VEGFA, HIF1A, ANGPT2 and FAK genes expression. On the contrary, hsa-miR-578 mimic transfection determined only a reduction of ANGPT2 transcript levels (Figure 5D).

## DISCUSSION

No tumor can grow beyond 100-200 μm without a blood supply which ensures the delivery of nutrients and oxygen to the malignant tissues [22]. The role of miRNAs as regulators of breast cancer angiogenesis has been well-indicated [4] but no data are available about their impact on familial breast cancer in this respect. The aim of the present study was to investigate whether signaling pathways related to angiogenesis in familial breast tumors could be affected by epigenetic regulation with respect to BRCA mutational status. Our previous study reported increased levels of angiopoietins and VEGF in tumor tissue of BRCA1/2 carriers, suggesting their contribution in blood vessels sprouting in this familial breast cancer subgroup [17]. Besides its role in maintaining the genomic stability, BRCA1 is also involved in neovascularization [13]. Next to our previous study [17], the expression of angiogenic and hypoxia-related markers has been previously investigated in breast cancer with respect to BRCA status [18-21]. Given miRNAs ability to regulate genes expression at post-transcriptional level [3], this is the first report exploring the impact of miRNAs deregulation on vasculature network within familial breast cancer. Recently, a functional link between BRCA1 and miRNAs has been described [6,12] but few reports are available about miRNA profiling in familial breast tumors also with respect to BRCA status [8-11]. Our analysis highlighted a set of 16 deregulated miRNAs between BRCA1/2-related and BRCAX tumors, almost all up-regulated in the former group with the exception of let-7i_star, miR-122, miR-578 and miR-573. As a single miRNA can target multiple transcripts and the co-expression of several miRNAs could affect diverse cellular signals [23], pathway enrichment analysis was used to provide insight into signals affected by deregulated miRNAs within familial breast tumors. The VEGF, HIF-1 and Focal Adhesion pathways were more deeply investigated for our purpose. Whereas VEGF [17-19] and HIF-1 alpha [18-21] expression has been investigated in BRCA-related tumors, to our knowledge no evidence is available about FAK. It is a non-receptor tyrosine kinase that, following the activation by both integrins and growth factors signals, can regulate several cell processes including angiogenesis [24]. The potential role of FAK in BRCA-related breast tumors still remains less investigated although BRCA1 has been described to be implicated in the invasion of breast cancer cells by controlling the turnover of specific receptors involved in focal adhesion, cell-cell and cell-matrix contacts [25]. Whereas FAK elevated levels and gene amplification have been well-demonstrated in breast cancer [26-28] and in the triple negative subtypes [29] respectively, only recently the association between tumor endothelial-FAK expression and breast cancer subtypes has been explored [30]. Interestingly the VEGF, Focal Adhesion and HIF-1 signaling pathways seemed to be affected by miR-122, miR-573 and miR-578, all three down-regulated in BRCA1/2-related tumors of the training set. Computational analysis revealed VEGFA and FAK as predictive targets of miR-578 and miR-573, whereas HIF1A was found as putative target of only miR-578. Given the pro-angiogenic and hypoxic properties of VEGFA, FAK and HIF1A, these data suggest an oncosuppressor role for both miR-578 and miR-573. Our observation was in line with a previous study on melanoma in which miR-573 exerted its oncosuppressor activity by targeting the melanoma cell adhesion molecule (MCAM) [31]. On the contrary, the implication of miR-578 in human tumor pathogenesis is still unclear. As VEGFA, HIF1A and FAK higher levels were observed in BRCA1/2 carriers, we supposed that miR-578 and miR-573 down-regulation could improve blood vessels sprouting by affecting VEGF, Focal Adhesion and HIF-1 signaling pathways. Our hypothesis was supported by a negative correlation found not only between all three pro-angiogenic genes with miR-578, but also between VEGFA and miR-573. Since anti-angiogenic strategies that directly target VEGF pathway have been well-described and many agents have been included in anti-angiogenesis trials [32], synthetic miR-573 and miR-578 mimics could be a possible promising novel strategy to inhibit blood vessels sprouting in BRCA-related breast tumors. A number of studies highlighted VEGF signaling cross-talk with integrin receptors as a critical event in the control of vascular permeability during the angiogenic switch [33, 34]. In addition, it has been shown that FAK can be an up-stream regulator of HIF1 alpha expression [35] and in turn, its phosphorylation can be enhanced by hypoxia [36]. The VEGF, FAK and HIF1 pathways cross-talk led us to suppose a key role of miR-573 and miR-578 deregulation in BRCA-related tumors angiogenesis.

In our previous study, the role of angiopoietin 2 in BRCA1/2-related breast cancer angiogenesis has been highlighted [17]. Recently, the capacity of angiopoietin 2 in regulating angiogenesis also in an integrin-dependent way through FAK phosphorylation has been reported [37]. Since computational analysis gave ANGPT2 as a putative target of both miR-578 and miR-573, we explored its expression in our validation set. As well as observed for VEGFA, FAK and HIF1A, also ANGPT2 higher levels have been detected in BRCA1/2-related tumors. We investigated the regulation of these genes expression by miR-573 and miR-578 in an *in vitro* model. Because of HIF1A, VEGFA, FAK and ANGPT2 genes did not result to be directly targeted by miR-573 or miR-578, the possibility of an indirect regulation was explored. Hsa-miR-578 mimic transfection in HEK293cells lead to HIF1A, VEGFA and ANGPT2 genes expression reduction suggesting an indirect regulation by this miRNA. Interestingly, by comparing SUM149PT and MCF-7 transfected breast cancer cells, a reduction of pro-angiogenic VEGFA and ANGPT2 mRNA level was observed only in BRCA1 deficient cells transfected by hsa-miR-573 mimic. Since higher levels of both genes were also reported in our previous study [17] we supposed that increased VEGFA and ANGPT2 expression levels found in BRCA1/2 related breast tumors could be also due to the above mentioned miRNAs aberration. Again, a key role played by VEGFA and angiopoieitn 2 in the neovascularization of BRCA1/2 related breast cancers has been highlighted. For the first time, miRNA-mediated angiogenesis was investigated in familial breast cancer with respect to BRCA mutational status. These data need further investigation in a larger cohort but our evidence highlights miR-578 and miR-573 involvement in BRCA1/2-related angiogenesis by affecting VEGF, FAK and HIF-1 signaling pathways.

## Materials and methods

### Patients

Tumor tissue samples were obtained from patients with a diagnosis of breast cancer and eligible for BRCA1/BRCA2 testing in accordance with previously-reported criteria [38]. All patients were enrolled at the IRCCS “Giovanni Paolo II” of Bari and followed in the Genetic Counselling Program of that Institute. All patients signed an informed consent form authorizing the research and all data have been processed with respect for privacy and anonymity. DNA from peripheral blood was screened for all BRCA1 and BRCA2 gene mutations as previously reported [39] and the familial breast tumors without BRCA1 and BRCA2 pathogenic mutations were defined as BRCAX breast cancers. The tumor tissue collection used for microarray analysis included 43 familial breast cancers stratified into 22 BRCA1/2 carriers and 21 BRCAX tumors. Validation analysis were performed on an independent set consisting of 19 breast tumors with a family history (8 BRCA1/2-related breast cancers and 11 BRCAX tumors). Moreover, for validation set the counterpart normal tissues from a representative number of patients (BRCA 1/2 carriers and BRCAX) were utilized as a control.

### MiRNA expression profiling

Total RNA, including microRNAs, was extracted from formalin-fixed, paraffin-embedded (FFPE) breast cancer samples using the RNeasy® FFPE Kit (QIAGEN) as per the manufacturer's protocol. 500 ng of RNA of each sample was labelled using the 3DNA Array Detection FlashTagTM RNA Labeling Kit according to the manufacturer's instructions, and analyzed with the GeneChip miRNA v. 1.0 Array (Affymetrix). This contains 46,228 probes comprising 7,815 probe sets, and covers 71 organisms including 1100 human miRNAs derived from the Sanger miRBase and miRNA database v11 (April 15, 2008, http://microrna.sanger.ac.uk). Firstly, poly (A) tailing was carried out at 37°C for 15 min in a volume of 15 ml reaction mix, which contained 1X Reaction Buffer, 1.5 ml MgCl_2_ (25 mM), 1 ml ATP Mix diluted 1:500, and 1 ml PAP enzyme. Secondly, Flash Tag Ligation was performed at room temperature for 30 min by adding 4 ml of 5X Flash Tag Ligation Mix Biotin and 2 ml T4 DNA Ligase into the 15 ml of reaction mix. To stop the reaction, 2.5 ml of Stop Solution was added. Each sample was hybridized on the array, washed, and stained with the Affymetrix Fluidics Station 450. They were then scanned with the Affymetrix GeneChip Scanner 3000 7G using the Command Console software (Affymetrix).

Raw data were normalized through the Robust Multi-array Average (RMA) method to remove systematic variations. Briefly, RMA corrects raw data for background using a formula which is based on a normal distribution and uses a linear model to estimate values on a log-scale. RMA normalization was performed using the “affy” package of the Bioconductor suite (http://www.bioconductor.org/) for the R statistical language (http://cran.r-project.org/). The default settings were used. Normalized values were statistically analyzed with MeV software v.4.8.1. Differentially-expressed miRNAs were detected through the t-test, and data were considered statistically significant when p<0.01. Microarray dataset has been deposited at ArrayExpress database under the accession number E-MTAB-2705.

### MiRNA validation by qRT-PCR

MiRNA expression analysis was performed on an independent set of familial breast tumors stratified into 8 BRCA1/2 carriers and 11 BRCAX samples.

Total RNA was extracted from FFPE breast tissues, as described above. The concentration of the isolated RNA was measured by a NanoDrop 8000 Spectrophotometer v2 1.0 (Thermo Scientific). Briefly, for detection of miR-573, miR-578 and miR-122 expression levels, 10 ng of total RNA were reverse transcribed using the TaqMan^®^ MicroRNA Reverse Transcription Kit using miRNA specific primers according to the manufacturer's protocol (Applied Biosystems). Real Time PCR analysis was performed on the ABI Prism 7000 Sequence Detection System (Applied Biosystems) using 3 μl of RT products in a reaction mixture containing TaqMan miRNA assay and the TaqMan Universal PCR Master Mix, according to the manufacturer's instructions (Applied Biosystems). All PCR reactions were performed in triplicate including no-template controls. Relative quantities of each miRNA were calculated using the ΔΔCt method after normalization with endogenous reference RNU 48.

### FAK, VEGF, HIF1A and ANGPT2 mRNA detection by qRT-PCR

For FAK (PTK2), VEGFA, HIF1A and Angiopoietin 2 (ANGPT2) gene expression analysis in the validation set (8 BRCA1/2 carriers and 11 BRCAX samples), 400 ng of total RNA were reverse transcribed in 20 μl using the High Capacity cDNA Reverse Transcription Kit, according to the manufacturer's protocol (Applied Biosystem). Quantitative real-time PCR was performed using 80 ng of cDNA in a final volume of 20 μl according to the manufacturer's instructions (Applied Biosystems), on the ABI Prism 7000 Sequence Detection System (Applied Biosystems). The ID assays used were the following: human FAK (Hs01056457_m1), human VEGFA (Hs00900055_m1), human HIF1A (Hs00153153_m1) and human ANGPT2 (Hs01048042_m1). RN18S1 (Hs03928985_g1) was used as the endogenous reference. Relative expression was calculated using the comparative Ct method. All PCRs were performed in triplicate including no-template controls.

Data analysis was performed using the GraphPad Prism statistics software package (GraphPad Prism 5.0). Statistical significance was determined using the Student's *t* test and the two-tailed Fischer's exact test. Pearson's correlation coefficient, r, was used to describe the association between miRNAs and their targets. Values of *p*≤0.05 were considered statistically significant.

### Dual-luciferase reporter assay and constructs

The Firefly luciferase-UTR reporter plasmid was constructed by introducing the 3′-UTR of VEGFA (NM_ 001025366.2), FAK (PTK2) (NM_005607.4), ANGPT2 (NM_001147.2), and HIF1A (NM_001530.3) genes into pmiR-REPORT miRNA Expression Reporter Vector System (Life Technology). The 3′-UTR sequence of the four analysed genes was amplified by PCR from HEK 293 cDNA. For ANGPT2 two different constructs were made, pMIR-ANGPT2-573 containing the hypothetical seed for *hsa-miR-573* and pMIR-ANGPT2-578 for *hsa-miR-578*, respectively. All constructs were verified by sequencing.

The reporter construct (50 ng), pSV-Renilla (2 ng, pRNL-SV40, Promega), and *hsa-miR-573* or *hsa-miR-578* mimic, or miRNA negative control (Sigma) were transfected into HEK293 cells using Lipofectamine 2000 (Life Technologies). Two different amount of miRNA mimic (1 and 2 pmoles) were used. After 48 hours, the cells were lysed in passive lysis buffer and assayed for both firefly and renilla luciferase activity using the Dual-GLO® Luciferase Assay System (Promega). Firefly luciferase activity was normalized to Renilla luciferase activity for each transfected well. Values are the mean ± S.E.M. of three experimental replicates from three independent transfections. Significance was determined by a two-tailed unpaired t test for means.

### FAK, VEGF, HIF1A and ANGPT2 genes expression in HEK293, MCF-7 and SUM149PT cell lines

Kidney HEK293 cells, BRCA1-proficient MCF-7 and BRCA1-deficient SUM149PT breast cancer cells were seeded in 12 well dishes and 24 h later were transfected with 20pmol of hsa-miR-578, hsa miR-573 and negative control miRNA mimic (HMI0800, HMI0793, and HMC0002, Sigma) using Lipofectamine 2000 (Life Technologies). Two days later, these cells were harvested and RNA was extracted using RNeasy Mini kit (Qiagen). Subsequently, total RNA was reverse transcribed for detection of genes, as described above. Moreover, quantitative real-time PCR was performed and relative expression levels of mRNAs were calculated as described above.
